# Eukaryotic Translation Elongation Factor 1A (eEF1A) Domain I from *S. cerevisiae* Is Required but Not Sufficient for Inter-Species Complementation

**DOI:** 10.1371/journal.pone.0042338

**Published:** 2012-07-30

**Authors:** Sandra Eltschinger, Eva Greganova, Manfred Heller, Peter Bütikofer, Michael Altmann

**Affiliations:** 1 Institute of Biochemistry & Molecular Medicine, University of Bern, Bern, Switzerland; 2 Swiss Tropical and Public Health Institute, Basel, Switzerland; 3 Mass Spectrometry and Proteomics, Department of Clinical Research, University Hospital, Bern, Switzerland; The John Curtin School of Medical Research, Australia

## Abstract

Ethanolamine phosphoglycerol (EPG) is a protein modification attached exclusively to eukaryotic elongation factor 1A (eEF1A). In mammals and plants, EPG is linked to conserved glutamate residues located in eEF1A domains II and III, whereas in the unicellular eukaryote *Trypanosoma brucei*, only domain III is modified by a single EPG. A biosynthetic precursor of EPG and structural requirements for EPG attachment to *T. brucei* eEF1A have been reported, but nothing is known about the EPG modifying enzyme(s). By expressing human eEF1A in *T. brucei*, we now show that EPG attachment to eEF1A is evolutionarily conserved between *T. brucei* and *Homo sapiens*. In contrast, *S. cerevisiae* eEF1A, which has been shown to lack EPG is not modified in *T. brucei.* Furthermore, we show that eEF1A cannot functionally complement across species when using *T. brucei* and *S. cerevisiae* as model organisms. However, functional complementation in yeast can be obtained using eEF1A chimera containing domains II or III from other species. In contrast, yeast domain I is strictly required for functional complementation in *S. cerevisiae*.

## Introduction

Eukaryotic translation elongation factor 1A (eEF1A) is a G-protein which delivers aminoacyl-tRNAs (aa-tRNAs) to the ribosomal A-site and allows for proper codon-anticodon mediated deciphering of the genetic code. This reaction requires hydrolysis of GTP to GDP and is assisted by eEF1B, a guanine nucleotide exchange factor that regenerates GTP-bound eEF1A and in *S. cerevisiae* consists of two subunits, eEF1Balpha which interacts directly with eEF1A and eEF1Bgamma. A third subunit, eEF1Bdelta, which also binds to eEF1A has been identified in other organisms such as *Artemia salina*, rabbit and human fetal cells (for a recent review describing eEF1 complexes, see [1]). The primary structure of eEF1A is highly conserved among all eukaryotes and prokaryotes (for which it is termed EF-Tu). The crystal structure of yeast eEF1A has been elucidated, it consists of three domains termed I, II and III [2], [3]. Whereas domain I binds guanine nucleotides, domains II and III are involved in the binding of aminoacyl-tRNAs [2] and eEF1Balpha binds in the hydrophobic pocket between domains I and II [3].

Being one of the most abundant proteins in eukaryotes (up to 5% of total cytosolic protein), several other functions besides its canonical role in translation elongation have been assigned to eEF1A (reviewed by [4]). Several covalent modifications such as phosphorylation [5], [6], lysine methylation [7], [8] and carboxy terminal methyl-esterification [9] have been reported to influence eEF1A’s biological activity. Furthermore, eEF1A is uniquely modified by ethanolamine phosphoglycerol (EPG) which is attached to conserved glutamic acid residues in domains II and III of mammalian and plant eEF1A [10], [11], [12]. In the parasitic protozoan *T. brucei,* only domain III of eEF1A is EPG-modified (residue E362) despite the presence of a second potential EPG modification site in domain II (residue E289) [13]. Previous studies using *T. brucei* as a model organism have shown that the ethanolamine moiety in EPG originates from the phospholipid phosphatidylethanolamine [13]. Moreover, E362 in *T. brucei* eEF1A is strictly required for attachment of EPG, indicating that the enzyme-mediated attachment of EPG (or its precursor molecule) is highly specific for glutamic acid at this position [14]. Surprisingly, *T. brucei* parasites expressing EPG-deficient eEF1A show no detectable growth defect demonstrating that, at least in cell culture, EPG attachment is not essential for eEF1A function in *T. brucei* [15]. Remarkably - and despite structural conservation of the glutamic acid residue in domain III (residue E372) - *S. cerevisiae* eEF1A may lack EPG: analysis of the peptide containing the postulated EPG attachment site by amino acid sequencing showed no evidence for the presence of EPG-modified glutamate [16]. We now confirm this observation using mass spectrometry. In addition, we show that in contrast to human eEF1A, *S. cerevisiae* eEF1A is not EPG-modified in *T. brucei*. Furthermore, we found that despite highly conserved protein sequences and similar predicted 3D-structures of yeast, *T. brucei* and mammalian eEF1A, orthologs cannot replace the endogenous protein. However, using plasmid shuffling techniques in *S. cerevisiae,* we were able to show that inter-species chimeric forms of eEF1A are able to functionally complement *in vivo*.

## Methods

### Materials

Unless otherwise specified, all reagents were of analytical grade and were from Merck (Darmstadt, Germany), Sigma-Aldrich (Buchs, Switzerland) or ICN Biomedicals (Tägerig, Switzerland). DNA polymerase was obtained from Invitrogen (Basel, Switzerland). Restriction enzymes were purchased from Thermo Scientific (St. Leon-Rot, Germany) or New England Biolabs (Ipswich, MA). [1-^3^H]ethan-1-ol-2-amine hydrochloride ([^3^H]Etn, 60 Ci mmol^−1^) was purchased from American Radiolabeled Chemicals Inc. (St. Louis, MO). Kodak MBX films were from Kodak SA (Lausanne, Switzerland).

### Generation of Expression Plasmids for Studies in T. Brucei

Plasmids for constitutive expression were derived from the trypanosome expression vector pCorleone [17]. The open reading frames (ORFs) of *H. sapiens* eEF1A (accession number NM_001402, position 64-1452, GenBank) and *S. cerevisiae* eEF1A (YPR080W, Saccharomyces Genome Database) were amplified using the primer pairs HA-1402f/1402r and HA-080Wf/080Wr, respectively (Table S1a), and subcloned between the XhoI and BamHI sites of pBluescript. Afterwards, the HindIII sites of both ORFs were destroyed using the QuikChange® Site-Directed Mutagenesis kit (Stratagene, Basel, Switzerland) and primer pairs Hs1402-895Af/Hs1402-895Ar and ScTEF1-411Ef/ScTEF1-411Er (Table S1a), respectively. Finally, the mutated ORFs were cloned between the HindIII and BamHI sites of pCorleone. The resulting vectors were used as template for the amplification of the appropriate ORFs lacking the HA-tags using primer pairs 1402f/1402r for *H. sapiens* eEF1A and 080Wf/080Wr for *S. cerevisiae* eEF1A. For conditional expression, PCR products were cloned between BamHI and HindIII restriction sites of pALC14 vector carrying a blasticidin resistance gene. The synthesis of the *Leishmania major* eEF1A ORF (accession number LmjF17.0080, TriTrypDB) was done using primer pair Lm0080f/Lm0080r (Table S1a). The PCR product was cloned into pALC14 vector, as described above. Prior to transfection into *T. brucei*, all vectors were linearized with NotI and SalI.

### Cell Culture and Transfection into T. Brucei

*T. brucei* procyclic form (PCF) Δprocyclin#1 (EP/GPEET null mutant) [18] and the derived cell lines were grown at 27°C in DTM supplemented with 15% heat-inactivated fetal bovine serum (FBS) (Invitrogen, Basel, Switzerland) [19]. The *T. brucei* PCF “C5” RNAi cell line against *T. brucei* eEF1A [20] derived from strain 29-13 [21] was cultured at 27°C in SDM79 supplemented with 15% FBS and 25 μg/ml hygromycin, 15 μg/ml G-418 and 2 μg/ml puromycin. The *T. brucei* strain C5-E conditionally expressing an ectopic copy of *T. brucei* eEF1A [20] was cultured in the same medium with additional 10 μg ml^−1^ blasticidin S HCl (Invitrogen). Trypanosomes were stable transfected using 10–15 μg of linearized plasmid DNA and selected with 10 μg ml^−1^ blasticidin S HCl. Transfection, selection with antibiotics and cloning were performed as described elsewhere [22]. RNA interference (RNAi) to down-regulate endogenous eEF1A expression and conditional expression of eEF1A proteins in the C5 cell line were induced by the addition of 1 μg/ml tetracycline [23].

### In vivo Labeling, Extraction and Immunoprecipitation

Trypanosomes were labeled during exponential growth (0.5−0.8×10^7^ cells ml^−1^) with 1 μCi ml^−1^ [^3^H]Etn for 18 h [24] and extracted as described previously [15]. The cell lysate was homogenized by passing three times through a 27-gauge needle and centrifuged at 16′000 g for 30 min. Subsequently, anti-HA Affinity Matrix (Roche Diagnostics) was added to the supernatant and incubated on a rotating device overnight at 4°C. The beads were spun down and washed three times with lysis buffer as described [15]. Tagged proteins were removed form the matrix by boiling in electrophoresis sample buffer.

### SDS-polyacrylamide Gel Electrophoresis (SDS-PAGE) and Immunoblotting

Extracted proteins were separated by glycine-SDS-PAGE under reducing conditions using 12% polyacrylamide gels. For fluorography of ^3^H-labeled proteins, gels were fixed (10% methanol, 7% acetic acid), soaked in Amplify (GE Healthcare), dried, and exposed to Kodak MBX films at −70°C. Semi-dry blotting was performed as previously described [15]. Mouse monoclonal antibody against hemagglutinin (α-HA; Covance, Berkeley, CA) was used at a dilution of 1∶3000 and was detected with secondary rabbit anti-mouse IgG antibody conjugated to horseradish peroxidase (Dako, Baar, Switzerland) at a dilution of 1∶5000, followed by enhanced chemiluminescence (Pierce, Lausanne, Switzerland).

### Mass Spectrometry

Immunoprecipitated HA-eEF1A proteins were analyzed by SDS-PAGE and immunoblotting as described above. After staining with Coomassie Brilliant Blue, HA-eEF1A proteins were cut out, cut into small pieces and exposed to on-membrane reductive alkylation and trypsin digestion [25]. The tryptic peptides were analyzed by liquid chromatography tandem mass spectrometry (nano-LC-MS/MS) as described previously [15]. Carboxy-terminally His_6x_-tagged ScEF1A was purified from yeast cell extracts essentially as described [26]. ScEF1A_His6x_ was transferred to a PVDF-membrane (BIO-RAD) and mass spectrometric analysis was carried out after on-membrane tryptic digestion. Tryptic peptides were analyzed by liquid mass spectrometry (nano-LC-MS/MS) as described previously.

### Northern Blot Analysis

Total RNA for Northern blotting was isolated using a SV Total RNA Isolation System (Promega, Madison, WI) and 10 μg of the total RNA was separated on formaldehyde-agarose gels (1% agarose, 2% formaldehyde in 20 mM 3-(N-morpholino)propanesulfonic acid). To control for equal loading, rRNA was visualized by ethidium bromide staining. Afterwards, RNA was transferred from the gel to positively charged nylon membrane, Hybond-N^+^ (GE Healthcare, Glattbrugg, Switzerland). Detection of polycistronic TbEF1A mRNA using a ^32^P-labeled probe made by random priming of the PCR product of TbEF1A intergenic region 1, subsequent hybridization and analysis by autoradiography using Bio-Max MS film and a TransScreen-HE intensifying screen was performed as described previously [20].

### Reverse Transcription PCR Analysis

To demonstrate the presence of transcripts of Hs-EF1A, Sc-EF1A, Lm-EF1A, and Tb-EF1A reverse transcription-PCR (RT-PCR) was performed. Total RNA was extracted from trypanosomes 72 h after induction with tetracycline. Complementary DNA (cDNA) was produced from single-strand RNA according to the manufacturer’s guidelines (SuperScript® II Reverse Transcriptase and Oligo(dT)20 Primer, Invitrogen) using 1 μg of extracted total RNA. Amplification of cDNA was done in 30 cycles using primer pairs (Table S1b), specific for the eEF1A ORFs in *S. cerevisiae* (ScTEF1_RT_f/ScTEF1_RT_r), *H. sapiens* (HsTEF1_RT_f/HsTEF1_RT_r), *L. major* (Lm080_RT (f)/Lm080_RT (r)) or *T. brucei* (TbTEF1_RT_f/TbTEF1_RT_r; accession number Tb927.10.2100, TriTrypDB). Total RNA extract was used as a negative control and PCR products were analyzed by electrophoresis using 0.8% agarose gel.

### In Silico Analysis

To build three-dimensional models of eEF1A proteins, pdb formats were generated as previously described [27], [28], [29] or directly downloaded from Protein Database (www.pdb.org). Subsequently structural models have been drawn with PyMOL Molecular Graphics System (DeLano WL).

### Cloning, Plasmids and Strains for in vivo Complementation Assays in S. Cerevisiae

Yeast strains as well as plasmids used and generated in this study are listed in the Supplemental Information (Tables S3, S4). eEF1A genes from *T. brucei, L. major, H. sapiens* were PCR amplified from plasmids pRS314_HA-TbTEF1 (*E. coli* strain #1873) and pMC4_HsTEF1 (*E. coli* strain #2056), pMC4_LmTEF1 (*E. coli* strain #2057) respectively. *C. albicans* eEF1A (CaTEF1) and *A. thaliana* eEF1A (AtTEF1) domain III were PCR amplified from a *C. albicans* cDNA library (a gift from D. Sanglard, University of Lausanne) and an *A. thaliana* cDNA library (a gift from D. Rentsch, University of Bern). 5′BamHI and 3′SpeI restriction sites – or 5′BamHI and 3′SacI restriction sites for CaTEF1– flanking the ends of ORFs were introduced with the help of oligonucleotide primers during PCR (Table S2a). Amino- and carboxy-terminally His_6x_-tagged forms of ScTEF1 were also obtained by PCR, using primers carrying tag sequences (Table S2a). Chimeric forms of eEF1A were obtained by exchanging single domains of eEF1A from different eukaryotic sources followed by ligation with yeast eEF1A domains. Domain exchange was achieved by introduction of unique restriction sites at highly conserved amino acid sequence motifs, as determined by multiple sequence alignments using ClustalW2 algorithm. Between eEF1A domains I and II, a SpeI restriction site was introduced by site-directed mutagenesis and a BamHI restriction site between domain II and III (Table S2b). Constructs were verified by sequencing and transformed into the conditional lethal yeast strain TKY102 (kindly provided by T. G. Kinzy, University of Medicine and Dentistry of New Jersey) carrying deletions of TEF1 and TEF2 (both genes encoding eEF1A) and the plasmid <TEF2_URA3> (for genotypic details, see Table S4). After transformation, plasmid shuffling was carried out on 0.5 mg/mL 5-FOA (5-fluoro orotic acid) plates to counterselect for the loss of <TEF2_URA3>. Media, yeast and *E. coli* cell transformations were performed according to standard procedures.

### Phenotypic Characterization of Mutant eEF1A Forms in S. Cerevisiae

All constructs were transformed into the conditional lethal strain TKY102. Transformants were subjected to plasmid shuffling and analyzed for growth on 0.5 mg/mL 5-FOA-containing plates by incubating them for 3–8 days at 25°C or 30°C. Viable mutants were harvested from 5-FOA and grown in 2–5 mL overnight cultures of YPD at 30°C. Next day, cells were diluted to a starting OD_600_ of 0.2–0.5 and grown until an OD_600_ of 1–1.2 was reached. Cell samples were brought to an OD_600_ of 1 for spotting dilutions. Plates were incubated at 25°C, 30°C or 35°C for 2–3 days.

## Results

### HsEF1A, but not ScEF1A, is Modified with EPG in T. Brucei

**Figure 1:**
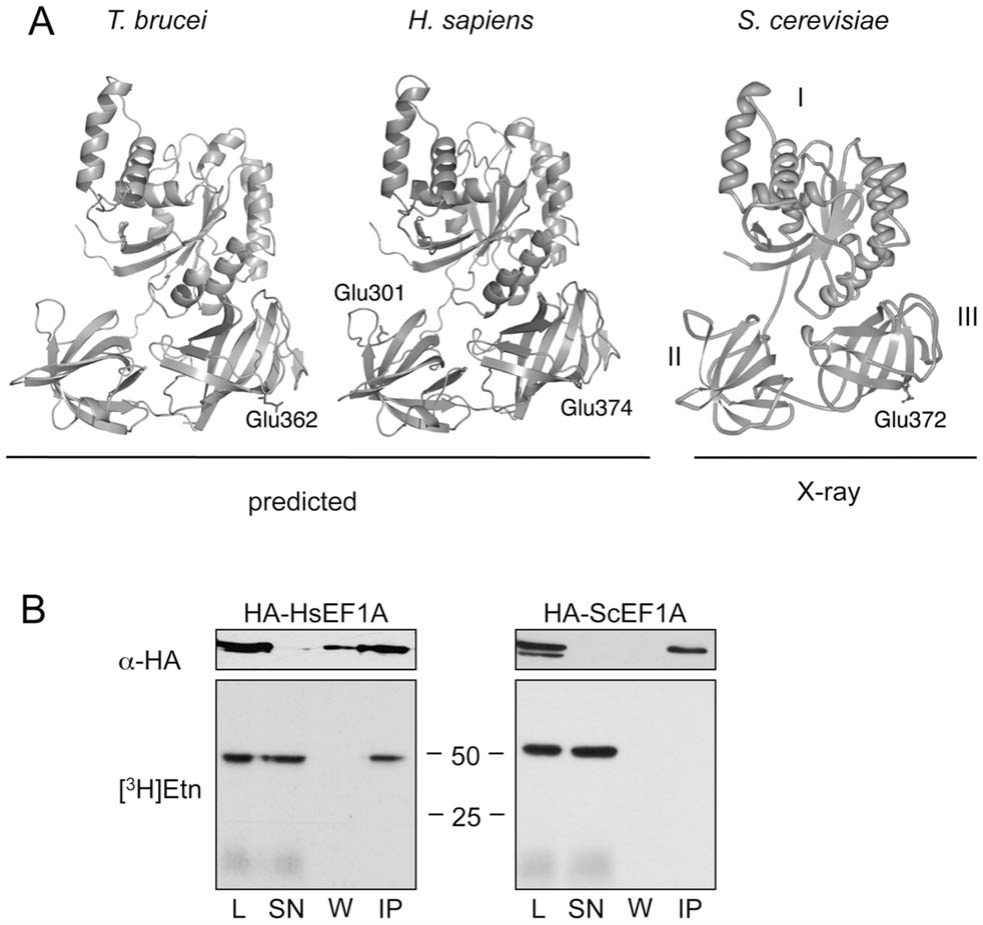
Expression and [^3^H]Etn-labeling of eEF1A orthologs in *T. Brucei.* (**A**) The predicted three-dimensional structures of eEF1A from *T. brucei* (left), *H. sapiens* (middle) and the X-ray structure of *S. cerevisiae* (right) are illustrated to document structural similarities. The positions of the glutamate residues representing potential EPG modification sites are indicated. The nomenclature of domains I, II, III is indicated for *S. cerevisiae* eEF1A. (**B**) *T. brucei* Δprocyclin#1 expressing HA-tagged human (HA-HsEF1A) or yeast (HA-ScEF1A) eEF1A were incubated in the presence of [^3^H]Etn for 18 h. Proteins in cell lysates (L) and in supernatants (SN), wash solutions (W) and the final pellet after immunoprecipitation using anti-HA antibody (IP) were separated by SDS-PAGE and analyzed by immunoblotting using α-HA monoclonal antibody (α-HA; upper panels) or fluorography (lower panels). Lanes contain extracts from 1×10^7^ (for L, SN and W) or 1.8×10^8^ cell equivalents (for IP). Molecular mass markers (kDA) are indicated.

eEF1A proteins from *H. sapiens*, *T. brucei* and *S. cerevisiae* show high amino acid sequence homology (Fig. S1 *)* and the predicted 3D structure of *H. sapiens* and *T. brucei* eEF1A closely match the three dimensional structure of *S. cerevisiae* eEF1A determined by X-ray crystallography [2], [3]. All structures predict the EPG attachment site of domain III to be at the surface of a β-sheet (Fig. 1A). Based on the (predicted) conservation of tertiary structures and EPG modification sites, we postulated that when expressed in *T. brucei* parasites, eEF1A of *S. cerevisiae* and *H. sapiens* would become modified by EPG by the trypanosome enzyme(s).

To test this hypothesis, HA-tagged *H. sapiens* eEF1A (HA-HsEF1A) and HA-tagged *S. cerevisiae* eEF1A (HA-ScEF1A) were expressed from ectopic copies in the EP/GPEET locus of *T. brucei* Δprocyclin#1 cells. Immunoblotting experiments demonstrated that HA-tagged eEF1A from both organisms were expressed in *T. brucei* and could be detected using α-HA antibodies (Fig. 1B, upper panels). Labeling of these parasites with [^3^H]Etn revealed a single radioactive band at 49 kDa after SDS-PAGE and fluorography, representing EPG-modified eEF1A (Fig. 1B, lower panels, lanes L) (see also [13], [15]). To distinguish between [^3^H]-labeled endogenous and HA-tagged eEF1A, parasite lysates were incubated with anti-HA antibody and the immunoprecipates were analyzed by SDS-PAGE and fluorography. The results show that while immunoprecipitated HA-HsEF1A was labeled with tritium, no radioactivity was recovered in HA-ScEF1A, indicating that human but not yeast eEF1A was modified with EPG (Fig. 1B, lower panels, lanes IP).

Mass spectrometry analysis of eEF1A proteins expressed in *T. brucei* confirmed the results obtained from *in vivo* labeling experiments. HA-HsEF1A revealed a tryptic fragment with *m/z* 804.386 and 402.697, representing the [M+H]^+^ and [M+2H]^2+^ ions of the modified peptide FAE*LK (with E* representing the potential EPG attachment site E374). Manual interpretation of the fragment spectra of both parental ions identified E374 as the site of EPG attachment (results not shown). In contrast, analysis of the HA-ScEF1A revealed a tryptic fragment with *m/z* 893.467 and 447.237, representing the [M+H]^+^ and [M+2H]^2+^ ions of the unmodified heptapeptide FDE*LLEK (with E* representing the potential EPG attachment site E372) (Table 1; see also [15]). MS/MS analysis of the parental ion at *m/z* 447.237 confirmed the absence of EPG (results not shown). The tryptic peptides SVEMHHEALSE*ALPGDNVGFNV (with E* representing E301 in HA-HsEF1A) and SVEMHHEQLE*QGVPGDNVGFNVK (with E* representing E298 in HA-ScEF1A) in domains II of the respective proteins were not modified with EPG either (see Table 1 for corresponding [M+H]^+^ and [M+2H]^2+^ ions). Together, these results demonstrate that despite the structural conservation of eEF1A proteins from different organisms, only HA-HsEF1A but not HA-ScEF1A could serve as substrate for EPG attachment in *T. brucei*.

**Table 1:** Characteristic ions of the tryptic fragments of eEF1A proteins detected by mass spectrometry.

Protein	Tryptic fragment	[M+H]^+^	[M+H]^2+^	[M+H]^3+^	EPG
T. *brucei* EF1A^a^	FAE*IESK/FAEIESK	1020.465/823.420^c^	510.736/412.214^d^	n.d.	+
*S. cerevisiae* EF1A^b^	FDE*LLEK/FDELLEK	893.467	−/447.23	n.d.	−
	FDE*LLEKNDR/FDELLEKNDR	n.d.	−/639.820	−/426.883	−
	FDE*LLEKNDRR/FDELLEKNDRR	n.d.	−/717.862	−/478.91	−
	SVEMHHEQLE*QGVPGDNVGFNVK/SVEM(ox)HHEQLEQGVPGDNVGFNVK	n.d.	−/1275.609 (M(ox)/1283.604)	−/850.741 (M(ox)/856.071)	−
*H. sapiens* EF1A^b^	FAE*LK/FAELK	804.386/−	402.697/−	n.d.	+
	SVEMHHEALSE*ALPGDNVGFNV/SVEMHHEALSEALPGDNVGFNV	n.d.	−/1240.595 (M(ox)/1248.592)	−/827.398 (M(ox)/832.73)	−
*S. cerevisiae* EF1AHis_6x_ c	FDE*LLEK/FDELLEK	n.d.	−/447.23	n.d.	−
	FDE*LLEKNDR/FDELLEKNDR	n.d.	−/639.820	−/426.882	−
	SVEMHHEQLE*QGVPGDNVGFNVK/SVEM(ox)HHEQLEQGVPGDNVGFNVK	n.d.	−/1275.605 (M(ox)/1283.603)	−/850.741 (M(ox)/856.07)	−

HA-eEF1A proteins expressed in *T. brucei* were purified, digested with trypsin and subjected to nano-LC-MS/MS as described in Materials and Methods. Purified carboxy-terminally His_6x_-tagged *S. cerevisiae* eEF1A was treated prior to nano-LC-MS/MS the same way as for HA-tagged eEF1A proteins. Tryptic fragments containing the site of potential EPG attachment E362, E298/E372, E301/E374 of domainII/domain III from *T. brucei*, *S. cerevisiae* and *H. sapiens* eEF1A, respectively (all marked with an asterisk) are shown with their corresponding [M+H]^+^, [M+H]^2+^ and [M+H]^3+^ ions. The last column indicates the presence (+) or absence (−) of EPG modifications based on ion data.

n.d., not detected.

−,not present.

Ox, oxidation.

a ,described in [15].

b ,expressed as HA-tagged protein in *T. brucei.*

c ,expressed as His_6x_-tagged protein in *S. cerevisiae.*

d ,the relative intensities of the [M+H]^+^ ions of the EPG-modified (*m/z* 1020.465) and unmodified (*m/z* 823.420) tryptic peptides suggest that >95% of *T. brucei* eEF1A is modified with EPG (see [15]).

To re-visit an earlier report showing that EPG is absent in *S. cerevisiae* eEF1A [16], we expressed a His_6x_-tagged form of eEF1A in a *S. cerevisiae* lacking endogenous eEF1A and analyzed the immunoprecipitated protein by LC-MS/MS. The results gained on the tryptic peptides FDELLEK and FDELLEKNDR showed that residue E372 was not modified either (Table 1). We conclude that *S. cerevisiae* eEF1A does not become EPG-modified in any of both tested systems (yeasts and trypanosomes).

### The Growth Defect of eEF1A-depleted T. Brucei cannot be Rescued by eEF1A Orthologs

**Figure 2:**
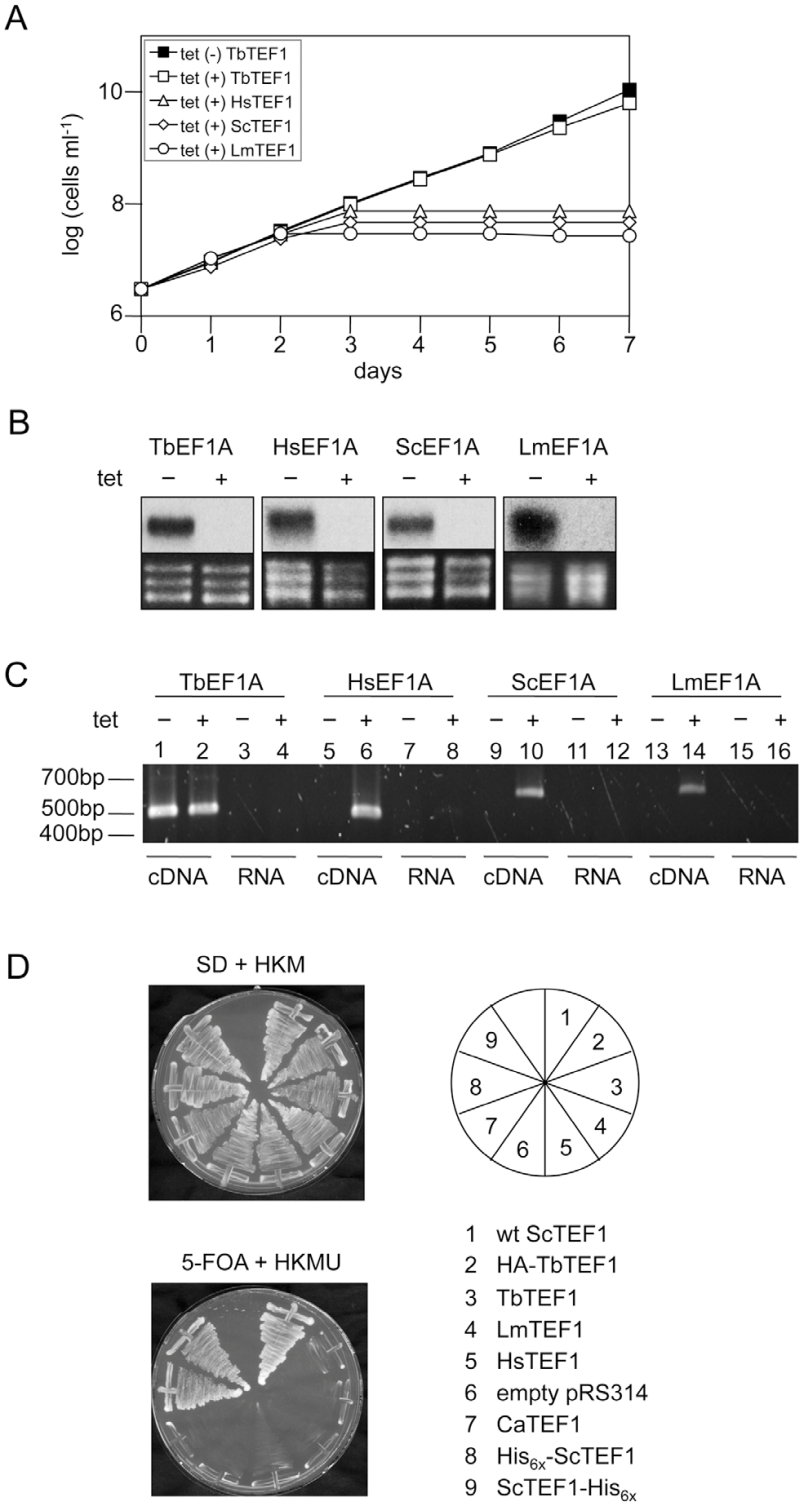
*In vivo* complementation assays in *T. brucei* and *S. cerevisiae* depleted for endogenous eEF1A. (**A**) *T. brucei* RNAi parasites expressing ectopic copies of TbEF1A, HsEF1A, ScEF1A or LmEF1A were cultivated in the absence (−) or presence (+) of tetracycline (tet) for 7 days. Each day, cultures were diluted to a cell density of 3×10^6^ cells/ml and incubated with fresh medium. Non-induced HsEF1A, ScEF1A and LmEF1A cell lines showed the same growth curve as non-induced cell line TbEF1A: for simplicity, only the growth curve for TbEF1A is shown (see also [20]). (**B**) Northern blots of total RNA extracted from parasites after 3 days of incubation in the absence (−) or presence (+) of tetracycline (tet) and hybridized with ^32^P-labeled probes against the intergenic region 1 of *T. brucei* eEF1A (top); rRNA was used as a loading control (bottom). (**C**) RT-PCR analysis of eEF1A transcripts. cDNA was synthesized from transcripts of *T. brucei* RNAi parasites cultured in the absence (−) or presence (+) of tetracycline for 72 h using primers specific for the different eEF1A orthologs (Table S1b). Lanes containing cDNA or total RNA (negative controls) are indicated. (**D**) Complementation assays in *S. cerevisiae* strain TKY102 expressing as unique source endogenous eEF1A from a URA3-plasmid. Cells were transformed with plasmids carrying genes encoding for different eEF1A orthologs. Upon transformation (upper panel), cells were incubated for several days on a plate containing 5-fluoroorotic acid (5-FOA) which is toxic in the presence of the URA3 plasmid. Only transformants which were able to loose due to mitotic segregation the URA3-plasmid grew on 5-FOA containing medium (lower panel). The numbers represent wild-type ScEF1A (1), HA-TbEF1A (2), TbEF1A (3), LmEF1A (4), HsEF1A (5), vector pRS314 (6, negative control), CaEF1A (7), His_6x_-ScEF1A (8), and ScEF1A-His_6x_ (9).

Based on the above mentioned observations, we decided to investigate if HsEF1A and ScEF1A are able to complement *T. brucei* eEF1A (TbEF1A) function. In a previous study, we generated a tetracycline-inducible *T. brucei* cell line (named C5) in which expression of TbEF1A was ablated using RNAi, resulting in growth arrest of the parasites [20]. In the same work, we showed that growth could be fully restored by expressing an inducible ectopic copy of TbEF1A. The same approach was used in the present work to introduce ectopic inducible copies of wild-type HsEF1A and ScEF1A into the C5 RNAi cell line and to study their possible functional complementation *in T. brucei*. The addition of tetracycline to the culture medium allowed us to simultanously down-regulate endogenous TbEF1A and induce expression of HsEF1A or ScEF1A. The results showed that neither HsEF1A nor ScEF1A were able to complement the growth defect of TbEF1A-depleted *T. brucei*, whereas TbEF1A fully restored growth (Fig. 2A, see also [20]). Complementation of TbEF1A was further studied by conditionally expressing eEF1A from *L. major* (LmEF1A) in the C5 cell line, which is highly homologous to TbEF1A, differing in only 25 amino acids (Fig. S1). However, no complementation was observed with LmEF1A (Fig. 2A).

Northern blot analyses using a probe against TbEF1A intergenic region 1 confirmed that in all cell lines transcription of endogenous TbEF1A was down-regulated (Fig. 2B, upper panels). To demonstrate that transcripts of the various eEF1A orthologs were made upon tetracycline induction, we performed RT-PCR using the same RNA samples used for Northern Blot analysis in combination with primer pairs allowing for discrimination between mRNA derived from endogenous TbEF1A versus that from eEF1A orthologs (Table S1b). RT-PCR analysis confirmed the absence of endogenous TbEF1A mRNA in the cell lines expressing HsEF1A, ScEF1A and LmEF1A, respectively (Fig. 2C, lanes 5, 9, 13). In addition, RT-PCR confirmed expression of ectopic TbEF1A, HsEF1A, ScEF1A and LmEF1A mRNA, i.e. the corresponding transcripts were detected after 72 h of induction (Fig. 2C, lanes 2, 6, 10, 14). Together, these results demonstrate that eEF1A orthologs are unable to restore normal growth of TbEF1A-depleted *T. brucei* in culture and fail to complement the essential functions of TbEF1A.

### eEF1A Orthologs Fail to Complement S. Cerevisiae eEF1A

Next, we decided to perform analogous complementation assays for *in vivo* functionality in *S. cerevisiae.* For this purpose, we cloned cDNAs encoding eEF1A-ORFs from *T. brucei, H. sapiens, C. albicans* and *L. major* into the yeast vector pRS314 under the control of the yeast eEF1A promoter und transformed them into the conditional lethal yeast strain TKY102, a conditionally lethal strain which expresses the essential yeast eEF1A activity from a <TEF2_URA3>plasmid [30]. Subsequently, transformed cells were subjected to the loss of the <URA3>plasmid by plating them on 5-FOA. As shown in Fig. 2D, amino- or carboxyterminal His_6x_-tagged forms of ScEF1A were able to replace the endogenous wild-type eEF1A gene copy by plasmid shuffling and allow for growth on 5-FOA. However, none of eEF1A orthologs was able to replace yeast eEF1A in TKY102 cells (Fig. 2D).

### Inter-species Chimeric Forms of eEF1A

**Figure 3:**
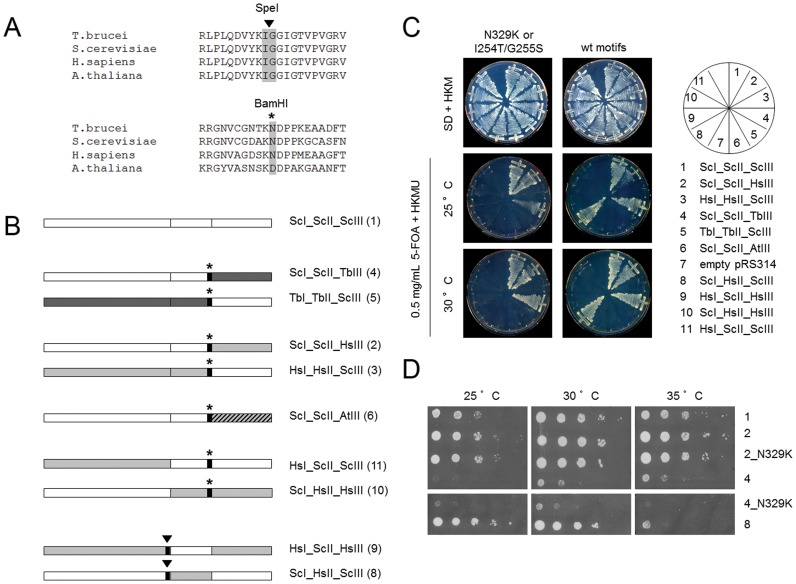
Complementation of chimeric eEF1A in *S. Cerevisiae.* (**A**) Sequence alignment of conserved amino acid motifs separating domains I and II (upper panel) and II and III (lower panel) of eEF1A from different sources. To generate chimeric constructs, synthetic SpeI (triangle) or BamHI (arrow) cloning sites were introduced. (**B**) Schematic representation of chimeric constructs. Arrows and triangles indicate the positions of the cloning sites which were removed by site-directed mutagenesis to reconstruct the original eEF1A sequences. Numbers in brackets correspond to clones shown in Fig. 3C. (**C**) Complementation of *S. cerevisiae* strain TKY102 with chimeric eEF1A constructs. Upper panels: Yeast cells growing on plates after transformation with different chimeric constructs. Middle and bottom panels: Counterselection for the loss of endogenous eEF1A on plates containing 5-FOA at 25 or 30°C. Left panels: Complementation assays with chimeric constructs carrying cloning sites causing a N329K mutation in the case of the artificial BamHI-site (separating domains II and III) or I254T/G255S mutations in the case of the artificial SpeI-site (separating domains I and II). Right panels: complementation assays with chimeric constructs after reconstructing wild type eEF1A sequence motifs. (**D**) Growth properties of *S. cerevisiae* complemented with chimeric eEF1A constructs. (1) positive control with yeast eEF1A; (2) Chimeric yeast constructs carrying human domain III - without or with N329K mutation; (4) Chimeric yeast constructs carrying *T. brucei* domain III - without or with N329K mutation; (8) Chimeric yeast eEF1A construct carrying humain domain II.

Since the results presented above demonstrate that full length eEF1A cDNAs from *H. sapiens, T. brucei, L. major* or *C. albicans* cannot complement for the loss of yeast or *T. brucei* eEF1A (Fig. 2), inter-species chimeric eEF1A genes were generated and analyzed in *S. cerevisiae*. Single domains of eEF1A from different eukaryotic species were interchanged. Introduction of restriction sites at highly conserved sequence motifs allowed for separation of single eEF1A domains (Fig. 3A). The “…KIGGI…” amino acid motif (residues 253–257 in ScEF1A) was mutagenized to introduce a SpeI restriction site between domains I and II. It is located at the first turn of the β sheet structure in domain II and is found in eEF1A from *T. brucei, H. sapiens* and *A. thaliana* (Fig. 3A; Fig. S1). This new restriction site creates a I254T/G255S mutation in eEF1A. The “…KNDP…” amino acid motif (residues 328 to 331 in ScEF1A) is conserved among all studied eEF1A orthologs (Fig. 3A; Fig. S1) and was mutagenized to introduce a BamHI restriction site between domains II and III. This new restriction site creates a N329K mutation in ScEF1A. Both mutations, I254T/G255S and N329K were re-converted by site-directed mutagenesis to the original eEF1A amino acid sequences following construction of the chimera. All constructs presented in Fig. 3B were transformed into the conditionally lethal yeast strain TKY102.

After plasmid shuffling, constructs carrying a non yeast domain III such as ScI_ScII_HsIII [construct 2] and ScI_ScII_TbIII [construct 4] were found to complement yeast strain TKY102, whereas constructs only carrying yeast domain III such as TbI_TbII_ScIII [construct 5] and HsI_HsII_ScIII [construct 3] did not complement, indicating that ScEF1A domain III may be interchangeable (Fig. 3C). However, no growth on 5-FOA was reported for ScI_ScII_AtIII [construct 6], neither at 25°C nor at 30°C.

As indicated, the point mutation created by introduction of an artificial BamHI site (N329K mutation) had no effect on *in vivo* functionality of ScI_ScII_HsIII [construct 2] and ScI_ScII_TbIII [construct 4] chimeric forms in *S. cerevisiae* (Fig. 3C). Growth studies on full media plates revealed that ScI_ScII_TbIII [construct 4] with or without N329K mutation rendered a severe slow growth phenotype, which was not observed for ScI_ScII_HsIII [construct 2] or ScI_ScII_HsIII [construct 2] N329K. Additionally, ScI_ScII_TbIII [construct 4] also showed a temperature sensitive growth phenotype (no growth at 35°C; Fig. 3D).

In addition, we observed that ScI_HsII_ScIII [construct 8] was able to complement endogenous yeast eEF1A. Although the amino acid sequence differs in only few residues from wild type ScEF1A, the construct produced a temperature sensitive growth phenotype (Fig. 3D). Interestingly, ScI_HsII_ScIII [construct 8] eEF1A chimera carrying a I254T, G255S double mutation due to introduction of the artificial SpeI restriction site was lethal (Fig. 3C). Also, chimeric construct ScI_HsII_HsIII [construct 10], which carries human domains II and III, was not able to complement for the loss of endogenous eEF1A.

Finally, all constructs lacking yeast domain I such as HsI_ScII_ScIII [construct 11], HsI_ScII_HsIII [construct 9], HsI_HsII_ScIII [construct 3] and TbI_TbII_ScIII [construct 5] were not able to support growth of TKY102 on 5-FOA plates (Fig. 3C, D).

## Discussion

Our results using in vivo labeling and mass spectrometry demonstrate that EPG is attached to E374 of HA-tagged HsEF1A expressed in *T. brucei* procyclic forms in culture. The attachment site corresponds to the same residue that is modified in HsEF1A in mammalian cells [12] and is conserved in TbEF1A [13] and plants [10]. In contrast, the second attachment site of HsEF1A, E301 was not modified in *T. brucei*. Thus, the enzymatic machinery of *T. brucei* EPG-modified HsEF1A the same way as endogenous TbEF1A, which is modified in domain II but not in domain III [15]. Since the enzymes involved in EPG attachment have not been characterized so far, we can only hypothesize if there is one phylogenetically ancient enzyme present in *T. brucei*, which later during evolution developed the capacity to modify domain II of eEF1A in mammalian and plant cells, or if two different EPG modifying enzyme systems exists in these multicellular organisms.

Interestingly, *S. cerevisiae* represents so far the only eukaryote lacking EPG modification of eEF1A ([16] and this work). It is not known, if yeast eEF1A lacks EPG because the biosynthetic pathway is deficient or if structure or sequence differences in yeast eEF1A prevent EPG attachment. We now found that HA-tagged *S. cerevisiae* eEF1A was not EPG-modified when expressed in *T. brucei*. The lack of EPG attachment was surprising, since the 3D-structure of yeast eEF1A closely matches that of *T. brucei* eEF1A and the (potential) EPG modification site on the surface of a β-sheet in domain III is conserved between yeast and *T. brucei*. In a previous report we have shown that replacement of the primary sequence around the EPG attachment site of *T. brucei* eEF1A (FAE*IESK; with E* representing the EPG attachment site) by the yeast sequence (FDE*LLEK; with E* representing the potential EPG attachment site) didn’t affect EPG modification in *T. brucei*. Thus, the different amino acid sequence around the EPG attachment site of *S. cerevisiae* eEF1A *per se* did not prevent EPG attachment [15].

Since EPG was only attached to human but not to yeast eEF1A despite structural similarity, we decided to perform complementation assays in *T. brucei* in which the endogenous (*T. brucei*) eEF1A is down-regulated by RNAi and the depletion phenotype is dependent on the expression of an eEF1A homolog from related (*L. major*) or unrelated organisms (*H. sapiens, S. cerevisiae*). The results showed that none of the conditionally expressed eEF1A proteins could rescue TbEF1A depletion in *T. brucei*. These findings were confirmed in eEF1A-depleted *S. cerevisiae* cells, where none of the expressed eEF1A proteins (*T. brucei, H. sapiens, L. major*, *C. albicans*) could rescue the depletion phenotype. The lack of complementation cannot be due to missing EPG modifications as human eEF1A is properly expressed and EPG-modified in *T. brucei*. Furthermore, it has been recently shown that a mutated version of *T. brucei* eEF1A lacking EPG is able to complement endogenous eEF1A in cell cultures [20]. Although the primary sequences and (predicted) three-dimensional structures of the different eEF1A orthologs tested in this study are highly homologous, it should be kept in mind that a single amino acid substitution may be sufficient to cause lack of complementation across species, e.g. by impeding proper interaction with partner proteins such as eEF1B, or by interfering with other essential posttranslational modifications. Furthermore, we cannot exclude that unequal codon usage among species may be a cause for the lack of complementation of the different eEF1A orthologs. Silent mutations can influence the folding of a protein due to varying levels of isoacceptor tRNAs affecting the velocity rate of translation elongation [31].

Together, these results demonstrate that the essential component of protein translation eEF1A, despite high sequence conservation and overall similarity in 3D-structure is unable to functionally complement across species. Interestingly, functional complementation across species has been reported for other translation factors such as eIF4E [32] but not between all species [33]. In addition, similar findings to those described here have been reported for another essential translation factor, eIF4A, in which orthologs from different sources could not support protein synthesis either *in vivo* or *in vitro* in an eIF4A-depleted yeast cell system [34], [35] despite the fact that they share sequence elements and function in large number of biochemical reactions ([36]; reviewed by [37]).

By constructing a series of inter-species eEF1A chimera and expressing them in the conditionally lethal strain *S. cerevisiae* TKY102, we found that the carboxy terminus of certain eEF1A proteins (comprising domain II or domain III) can be exchanged without loss of viability. Domain III from *A. thaliana* was not able to complement in conjunction with yeast eEF1A domains I and II as a chimeric construct. This came as a surprise, as plants are assumed to be evolutionary closer related to yeast than human or trypanosome genes and demonstrated that there are also constraints to exchanging the essential domain III of yeast eEF1A.

In contrast, the amino terminus (comprising domain I) is essential for function in *S. cerevisiae*. Similar findings have been reported for eubacterial EFTu, where recombinant chimeric elongation factor containing domain I of aEF1A from archea *Sulfolobus solfataricus* and domains II and III from *Escherichia coli* EF-Tu did not sustain poly(Phe) synthesis in either a *S. solfataricus* or a *E. coli* assay system [38].

The essentiality of domain I may be explained as follows: (i) Domain I is crucial for interactions with macromolecules involved in the process of protein elongation such as ribosomal proteins and other interaction partners like eEF1B. eEF1A is a well-known member of the superfamily of GTPases and carries regions essential for binding of GTP and the Mg^2+^ ion in domain I (switch regions 1 and 2). The GTPase activity of eEF1A is affected by ribosomes that trigger the hydrolysis of GTP in eukaryotes and similarly, they enhance by more than100-fold the intrinsic GTPase activity of EF-Tu in bacteria [39]. In addition, the dissociation of GDP from eEF1A is accelerated by eEF1B (EFTs in bacteria) that binds between domain I and domain II of eEF1A [40]–[41]. Upon binding of eEF1B conformation changes of eEF1A i.e. in the switch region 2 are induced and affect the nucleotide exchange [3], [41]. (ii) Domain I carries post-translational modifications such as phosphorylations and single-, di- and tri-methylations that often vary between species (reviewed by [7], [16], [42], [43]). These differences may affect the rate of binding of tRNA to ribosomes [6] or critical protein-protein interactions with other factors involved in protein synthesis such as translation elongation factor eEF1Balpha [3] or factors interacting with eEF1A involved in other functions than translation (reviewed in [4]). In a similar fashion, various (in)direct binding partners of translation initiation factor eIF4A such as eIF4G, p97, eIF4AIII and eIF4E have been reported to have different binding properties when comparing mammalian to yeast eIF4A [44], [45], [46], [47]. In conclusion, the functional evolution of translation factors such as eEF1A and eIF4A may be driven by structural changes in protein partners rather than by changes in its own amino acid sequence.

## Supporting Information

Figure S1 **ClustalW Multiple sequence alignment of eEF1A sequences from different sources.** Yellow indicates conserved and semi-conserved substitutions within a column of residues. Unconserved changes are black boxed. eEF1A domain I is shown underlined in blue, domain II underlined in orange and domain III underlined in red. Protein accession numbers for eEF1A sequences: *T. brucei,* P86934; *L. major*, Q4QEI8; *S. cerevisiae,* P50522; *C. albicans,* Q59K68; *H. sapiens,* P68104; *A. thaliana,* P1390.
(DOC)

Table S1 **Primers used for experiments with** ***T. Brucei.*** Table S1a. Primers for generation of constructs used for *in vivo* complementation assay in *T. brucei*. Underlined nucleotides indicate mutated triplets. Table S1b. Primers used in RT-PCR method to detect specific eEF1A transcripts in *T. brucei* RNAi cell line C5.
(DOC)

Table S2 **Primers used for experiments with** ***S. Cerevisiae*** **.** Table S2a. Primers used to produce ScEF1A_His6X_ and amplification of eEF1A genes from different eukaryotic sources. Underlined nucleotides indicate sequences of restriction sites, letters in bold prints the His_6x_-tag sequence. Table S2b. Primers to produce interspecies chimeric constructs. Underlined nucleotides indicate sequences of restriction sites, letters in bold represent mutagenized nucleotides.
(DOC)

Table S3 **Plasmids used for experiments with** ***S. Cerevisiae.***
(DOC)

Table S4 **Genotype of yeast strains used in this work.**
(DOC)
