# Supplementary material for: Eukaryotic Translation Elongation Factor 1A (eEF1A) Domain I from S. cerevisiae Is Required but Not Sufficient for Inter-Species Complementation
Source: PLoS One. 2012 Jul 30;7(7):e42338. doi: 10.1371/journal.pone.0042338 (PMC3408446; doi:10.1371/journal.pone.0042338)
Supplement: Figure S1 — ClustalW Multiple sequence alignment of eEF1A sequences from different sources. Yellow indicates conserved and semi-conserved substitutions within a column of residues. Unconserved changes are black boxed. eEF1A domain I is shown underlined in blue, domain II underlined in orange and domain III underlined in red. Protein accession numbers for eEF1A sequences: T. brucei, P86934; L. major, Q4QEI8; S. cerevisiae, P50522; C. albicans, Q59K68; H. sapiens, P68104; A. thaliana, P1390. (DOC) [file pone.0042338.s001.doc]

**Supporting Information**

**Figure S1**

T.brucei MGKEKVHMNLVVVGHVDAGKSTATGHLIYKCGGIDKRTIEKFEKEAADIGKASFKYAWVL 60

L.major MGKDKVHMNLVVVGHVDAGKSTATGHLIYKCGGIDKRTIEKFEKEAAEIGKASFKYAWVL 60

S.cerevisiae MGKEKSHINVVVIGHVDSGKSTTTGHLIYKCGGIDKRTIEKFEKEAAELGKGSFKYAWVL 60

C.albicans MGKEKTHVNVVVIGHVDSGKSTTTGHLIYKCGGIDKRTIEKFEKEAAELGKGSFKYACVL 60

H.sapiens MGKEKTHINIVVIGHVDSGKSTTTGHLIYKCGGIDKRTIEKFEKEAAEMGKGSFKYAWVL 60

A.thaliana MGKEKFHINIVVIGHVDSGKSTTTGHLIYKLGGIDKRVIERFEKEAAEMNKRSFKYAWVL 60

***:* *:*:**:****:****:******* ******.**:******::.* ***** **

T.brucei DKLKAERERGITIDIALWKFESPKSVFTIIDAPGHRDFIKNMITGTSQADAAILIIASAQ 120

L.major DKLKAERERGITIDIALWKFESPKSVFTIIDAPGHRDFIKNMITGTSQADAAILMIDSTH 120

S.cerevisiae DKLKAERERGITIDIALWKFETPKYQVTVIDAPGHRDFIKNMITGTSQADCAILIIAGGV 120

C.albicans DKLKAERERGITIDIALWKFETPKYHVTVIDAPGHRDFIKNMITGTSQADCAILIIAGGT 120

H.sapiens DKLKAERERGITIDISLWKFETSKYYVTIIDAPGHRDFIKNMITGTSQADCAVLIVAAGV 120

A.thaliana DKLKAERERGITIDIALWKFETTKYYCTVIDAPGHRDFIKNMITGTSQADCAVLIIDSTT 120

***************:*****:.* *:*********************.*:*:: .

T.brucei GEFEAGISKDGQTREHALLAFTLGVKQMVVCCNKMDDKTVNYGQERYDEIVKEVSAYIKK 180

L.major GGFEAGISKDGQTREHALLAFTLGVKQMVVCCNKMDDKTVTYAQSRYDEISKEVGAYLKR 180

S.cerevisiae GEFEAGISKDGQTREHALLAFTLGVRQLIVAVNKMDSVK--WDESRFQEIVKETSNFIKK 178

C.albicans GEFEAGISKDGQTREHALLAYTLGVKQLIVAVNKMDSVK--WDKNRFEEIIKETSNFVKK 178

H.sapiens GEFEAGISKNGQTREHALLAYTLGVKQLIVGVNKMDSTEPPYSQKRYEEIVKEVSTYIKK 180

A.thaliana GGFEAGISKDGQTREHALLAFTLGVKQMICCCNKMDATTPKYSKARYDEIIKEVSSYLKK 180

* *******:**********:****:*:: **** : : *::** **.. ::*:

T.brucei VGYNVEKVRFVPISGWQGDNMIEKSEKMPWYK------------GPTLLEALDMLEPPVR 228

L.major VGYNPEKVRFIPISGWQGDNMIEKSDNMPWYK------------GPTLLDALGMLEPPVR 228

S.cerevisiae VGYNPKTVPFVPISGWNGDNMIEATTNAPWYKGWEKETKAGVVKGKTLLEAIDAIEQPSR 238

C.albicans VGYNPKTVPFVPISGWNGDNMIEPSTNCPWYKGWEKETKSGKVTGKTLLEAIDAIEPPTR 238

H.sapiens IGYNPDTVAFVPISGWNGDNMLEPSANMPWFKGWKVTRKDGNASGTTLLEALDCILPPTR 240

A.thaliana VGYNPDKIPFVPISGFEGDNMIERSTNLDWYK------------GPTLLEALDQINEPKR 228

:*** ..: *:****::****:: : : *:* * ***:*:. : * *

T.brucei PSDKPLRLPLQDVYKIGGIGTVPVGRVETGVMKPGDVVTFAPANVTTEVKSIEMHHEQLA 288

L.major PVDKPLRLPLQDVYKIGGIGTVPVGRVETGIMKPGDVVTFAPANVTTEVKSIEMHHEQLA 288

S.cerevisiae PTDKPLRLPLQDVYKIGGIGTVPVGRVETGVIKPGMVVTFAPAGVTTEVKSVEMHHEQLE 298

C.albicans PTDKPLRLPLQDVYKIGGIGTVPVGRVETGIIKAGMVVTFAPAGVTTEVKSVEMHHEQLA 298

H.sapiens PTDKPLRLPLQDVYKIGGIGTVPVGRVETGVLKPGMVVTFAPVNVTTEVKSVEMHHEALS 300

A.thaliana PSDKPLRLPLQDVYKIGGIGTVPVGRVETGMIKPGMVVTFAPTGLTTEVKSVEMHHESLL 288

* ****************************::*.* ******..:******:***** *

T.brucei EATPGDNVGFNVKNVSVKDIRRGNVCGNTKNDPPKEAADFTAQVIILNHPGQIGNGYAPV 348

L.major EAQPGDNVGFNVKNVSVKDIRRGNVCGNSKNDPPKEAADFTAQVIVLNHPGQISNGYAPV 348

S.cerevisiae QGVPGDNVGFNVKNVSVKEIRRGNVCGDAKNDPPKGCASFNATVIVLNHPGQISAGYSPV 358

C.albicans EGVPGDNVGFNVKNVSVKEIRRGNVCGDSKNDPPKGCDSFNAQVIVLNHPGQISAGYSPV 358

H.sapiens EALPGDNVGFNVKNVSVKDVRRGNVAGDSKNDPPMEAAGFTAQVIILNHPGQISAGYAPV 360

A.thaliana EALPGDNVGFNVKNVAVKDLKRGYVASNSKDDPAKGAANFTSQVIIMNHPGQIGNGYAPV 348

:. ************:**:::** *..::*:**. . .*.: **::**** *. **:**

T.brucei LDCHTSHIACKFAEIESKIDRRSGKELEKAPKSIKSGDAAIVRMVPQKPMCVEVFNDYAP 408

L.major LDCHTSHIACRFAEIESKIDRRSGKELEKNPKAIKSGDAAIVKMVPQKPMCVEVFNDYAP 408

S.cerevisiae LDCHTAHIACRFDELLEKNDRRSGKKLEDHPKFLKSGDAALVKFVPSKPMCVEAFSEYPP 418

C.albicans LDCHTAHIACKFDTLVEKIDRRTGKKLEENPKFVKSGDAAIVKMVPTKPMCVEAFTDYPP 418

H.sapiens LDCHTAHIACKFAELKEKIDRRSGKKLEDGPKFLKSGDAAIVDMVPGKPMCVESFSDYPP 420

A.thaliana LDCHTSHIAVKFSEILTKIDRRSGKEIEKEPKFLKNGDAGMVKMTPTKPMVVETFSEYPP 408

*****:*** :* : * ***:**::*. ** :*.***.:* :.* *** ** *.:*.*

T.brucei LGRFAVRDMRQTVAVGIIKAVTKKDGSGGKVTKAAVKASKK- 449

L.major LGRFAVRDMRQTVAVGIIKGVNKKEGSGGKVTKAAAKASKK- 449

S.cerevisiae LGRFAVRDMRQTVAVGVIKSVDKTEK-AAKVTKAAQKAAKK- 458

C.albicans LGRFAVRDMRQTVAVGVIKSVEKSDK-AGKVTKAAQKAAKK- 458

H.sapiens LGRFAVRDMRQTVAVGVIKAVDKKAAGAGKVTKSAQKAQKAK 462

A.thaliana LGRFAVRDMRQTVAVGVIKSVDKKDPTGAKVTKAAVKKGAK- 449

****************:**.* *. ..****:* *
