# Supplementary material for: Eukaryotic Translation Elongation Factor 1A (eEF1A) Domain I from S. cerevisiae Is Required but Not Sufficient for Inter-Species Complementation
Source: PLoS One. 2012 Jul 30;7(7):e42338. doi: 10.1371/journal.pone.0042338 (PMC3408446; doi:10.1371/journal.pone.0042338)
Supplement: Table S1 — Primers used for experiments with T. Brucei. Table S1a. Primers for generation of constructs used for in vivo complementation assay in T. brucei. Underlined nucleotides indicate mutated triplets. Table S1b. Primers used in RT-PCR method to detect specific eEF1A transcripts in T. brucei RNAi cell line C5. (DOC) [file pone.0042338.s002.doc]

**TABLE S1a**

| **Primer** | **Direction** | **Sequence (5’🡪3’)** | **Gene Accession Number** |
| --- | --- | --- | --- |
| HA-1402f | forward | GAGCCCCTCGAGAAGCTTATGTACCCTTATGACGTACCAGACTATGCAATGGGAAAGGAAAAGACT | NM_001402 |
| 1402f | forward | GAGCCCAAGCTTATGGGAAAGGAAAAGACT | NM_001402 |
| 1402r | reverse | CGGGATCCTCATTTAGCCTTCTGAGC | NM_001402 |
| Hs1402-895Af | forward | ATGCACCATGAAGCGTTGAGTGAAGCTC | NM_001402 |
| Hs1402-895Ar | reverse | GAGCTTCACTCAACGCTTCATGGTGCAT | NM_001402 |
| HA-080Wf | forward | GCAGGACTCGAGAAGCTTATGTACCCTTATGACGTACCAGACTATGCAATGGGTAAAGAGAAGTCT | YPR080W |
| 080Wf | forward | GCAGGAAAGCTTATGGGTAAAGAGAAGTCT | YPR080W |
| 080Wr | reverse | GGAGCAGGATCCTTATTTCTTAGCAGCCTT | YPR080W |
| ScTEF1 411Ef | forward | CCAATGTGTGTTGAGGCTTTCAGTGAATAC | YPR080W |
| ScTEF1 411Er | reverse | GTATTCACTGAAAGCCTCAACACACATTGG | YPR080W |
| Lm0080f | forward | GCAGGAAAGCTTATGGGCAAGGATAAGGTG | LmjF17.0080 |
| Lm0080r | reverse | GGAGCAGGATCCTTACTTCTTCGAAGCCTT | LmjF17.0080 |

**TABLE S1b**

| **Primer** | **Direction** | **Sequence (5’🡪3’)** | **Gene Accession Number** |
| --- | --- | --- | --- |
| HsTEF1_RT_f | forward | ACCAGCAAGTACTATGTG | NM_001402 |
| HsTEF1_RT_r | reverse | CTGGCATTGCCATCCTTA | NM_001402 |
| ScTEF1_RT_f | forward | TGTCGTCAAGGGTAAGAC | YPR080W |
| ScTEF1_RT_r | reverse | AGATGGAACGAACTTGAC | YPR080W |
| Lm080_RT_f | forward | GCAGGAAAGCTTATGGGCAAGGATAAGGTG | LmjF17.0080 |
| Lm080_RT_r | reverse | GGAGCAGGATCCTTACTTCTTCGAAGCCTT | LmjF17.0080 |
| TbTEF1_RT_f | forward | GAAATCCGAGAAGATGCC | Tb927.10.2100 |
| TbTEF1_RT_r | reverse | CGACTCGATCACCGCGAA | Tb927.10.2100 |
