# Supplementary material for: Eukaryotic Translation Elongation Factor 1A (eEF1A) Domain I from S. cerevisiae Is Required but Not Sufficient for Inter-Species Complementation
Source: PLoS One. 2012 Jul 30;7(7):e42338. doi: 10.1371/journal.pone.0042338 (PMC3408446; doi:10.1371/journal.pone.0042338)
Supplement: Table S2 — Primers used for experiments with S. Cerevisiae . Table S2a. Primers used to produce ScEF1AHis6X and amplification of eEF1A genes from different eukaryotic sources. Underlined nucleotides indicate sequences of restriction sites, letters in bold prints the His6x-tag sequence. Table S2b. Primers to produce interspecies chimeric constructs. Underlined nucleotides indicate sequences of restriction sites, letters in bold represent mutagenized nucleotides. (DOC) [file pone.0042338.s003.doc]

**TABLE S2a**

| **Primer** | **Description** | **Direction** | **Sequence (5’🡪3’)** | | **Gene Accession Number** |
| --- | --- | --- | --- | --- | --- |
| OL848 | ScTEF1_5’BamHI | forward | CCGGATCCATGGGTAAAGAGAAGTCTC | | NM_001184177 |
| MA806 | ScTEF1-His6x_3’SpeI | reverse | GGACTAGTTTA**ATGGTGATGGTGATGGTG**TTTCTTAGCAGCCTTTTG | NM_001184177 | |
| MA805 | His_6x_-ScTEF1_5’BamHI | forward | CCGGATCCATG**CATCACCATCACCATCAC**GGTAAAGAGAAGTCTCAC | NM_001184177 | |
| MA589 | ScTEF1_3’SpeI | reverse | GGACTAGTTTATTTCTTAGCAGCCTT | | NM_001184177 |
| OL1263 | CaTEF1_5’BamHI | forward | ccggatccatgggtaaagaaaaaactcacgtt | | XM_705052 |
| OL1270 | CaTEF1_3’SacI | reverse | cccgagctcttatttcttagcagctttttgagc | | XM_705052 |
| OL771 | TbTEF1_5’BamHI | forward | CCGGATCCATGGGAAAGGAAAAGGTG | | XM_817373 |
| MA590 | TbTEF1_3’SpeI | reverse | GGACTAGTTTATTTCTTCGAAGCC | | XM_817373 |
| OL772 | LmTEF1_5’BamHI | forward | CCGGATCCATGGGCAAGGATAAGGTG | | XM_001682208 |
| OL773 | LmTEF1_3’SpeI | reverse | GGACTAGTTTACTTCTTCGAAGCCTTC | | XM_001682208 |
| OL774 | HsTEF1_5’BamHI | forward | CCGGATCCATGGGAAAGGAAAAGACTC | | NM_001402 |
| OL775 | HsTEF1_3’SpeI | reverse | GGACTAGTTCATTTAGCCTTCTGAGC | | NM_001402 |

**TABLE S2b**

| **Primer** | **Description** | **Direction** | **Sequence (5’🡪3’)** |
| --- | --- | --- | --- |
| OL794 | ScTEF1 dII🡪III_BamHI | forward | GGTGACGCTAAGAA**G**GATCCACCAAAGGGT |
| OL795 | ScTEF1 dII🡪III_BamHI | reverse | ACCCTTTGGTGGATC**C**TTCTTAGCGTCACC |
| OL796 | TbTEF1 dII🡪III_BamHI | forward | GGTAACACCAAGAA**G**GA**T**CCCCCAAAGGAGGCC |
| OL797 | TbTEF1 dII🡪III_BamHI | reverse | GGCCTCCTTTGGGGG**A**TC**C**TTCTTGGTGTTACC |
| OL798 | HsTEF1 dII🡪III_BamHI | forward | GGTGACAGCAAAAA**G**GA**T**CCACCAATGGAAGCA |
| OL799 | HsTEF1 dII🡪III_BamHI | reverse | TGCTTCCATTGGTGG**A**TC**C**TTTTTGCTGTCACC |
| OL1157 | ScII🡪HsIII_∆BamHI | forward | GGTGACGCTAAGAA**C**GA**C**CCACCAATGGAAGCA |
| OL1158 | ScII🡪HsIII_∆BamHI | reverse | TGCTTCCATTGGTGG**G**TC**G**TTCTTAGCGTCACC |
| OL1159 | ScII🡪TbIII_∆BamHI | forward | GGTGACGCTAAGAA**C**GA**C**CCCCCAAAGGAGGCC |
| OL1160 | ScII🡪TbIII_∆BamHI | reverse | GGCCTCCTTTGGGGG**G**TC**G**TTCTTAGCGTCACC |
| OL1161 | TbII🡪ScIII_∆BamHI | forward | GGTAACACCAAGAA**C**GATCCACCAAAGGGT |
| OL1162 | TbII🡪ScIII_∆BamHI | reverse | ACCCTTTGGTGGATC**G**TTCTTGGTGTTACC |
| OL1163 | HsII🡪ScIII_∆BamHI | forward | GGTGACAGCAAAAA**T**GATCCACCAAAGGGT |
| OL1164 | HsII🡪ScIII_∆BamHI | reverse | ACCCTTTGGTGGATC**A**TTTTTGCTGTCACC |
| OL1166 | AtTEF1_3’SpeI | reverse | GGACTAGTTCACTTGGCACCCTTC |
| OL1207 | AtTEF1dIII_5’BamHI | forward | CCGGATCCGACCCTGCCAAGGGTGCT |
| OL1169 | ScII🡪AtIII_∆BamHI | forward | GGTGACGCTAAGAA**C**GA**C**CCTGCCAAGGGTGCT |
| OL1170 | ScII🡪AtIII_∆BamHI | reverse | AGCACCCTTGGCAGG**G**TC**G**TTCTTAGCGTCACC |
| OL1171 | ScI🡪ScII_SpeI (ScI_ScII_HsIII) | forward | CAAGATGTTTACAAGA**C**T**A**GTGGTATTGGTACTGTG |
| OL1172 | ScI🡪ScII_SpeI (ScI_ScII_HsIII) | reverse | CACAGTACCAATACCAC**T**A**G**TCTTGTAAACATCTTG |
| OL1173 | ScI🡪HsII_∆SpeI (ScI_HsII_HsIII) | forward | CAAGATGTTTACAAGA**T**T**G**GTGGTATTGGTACTGTT |
| OL1174 | ScI🡪HsII_∆SpeI (ScI_HsII_HsIII) | reverse | AACAGTACCAATACCAC**C**A**A**TCTTGTAAACATCTTG |
| OL1175 | HsI🡪ScII_∆SpeI (HsI_ScII_ScIII) | forward | CAGGATGTCTACAAAA**T**T**G**GTGGTATTGGTACT |
| OL1176 | HsI🡪ScII_∆SpeI (HsI_ScII_ScIII) | reverse | AGTACCAATACCAC**C**A**A**TTTTGTAGACATCCTG |
| OL1177 | HsI🡪HsII_SpeI (HsI_HsII_ScIII) | forward | CAGGATGTCTACAAAA**C**T**A**GTGGTATTGGTACTGTT |
| OL1178 | HsI🡪HsII_SpeI (HsI_HsII_ScIII) | reverse | AACAGTACCAATACCAC**T**A**G**TTTTGTAGACATCCTG |
| OL1223 | ScII🡪HsIII_BamHI | forward | TGTGGTGACGCTAAGAAGGATCCACCAATGGAAGCAGCT |
| OL1224 | ScII🡪HsIII_BamHI | reverse | AGCTGCTTCCATTGGTGGATCCTTCTTAGCGTCACCACA |
| OL1225 | ScII🡪ScIII_∆BamHI (HsI_ScII_ScIII) | forward | TGTGGTGACGCTAAGAA**C**GATCCACCAAAGGGTTGC |
| OL1226 | ScII🡪ScIII_∆BamHI (HsI_ScII_ScIII) | reverse | GCAACCCTTTGGTGGATC**G**TTCTTAGCGTCACCACA |
| OL1227 | HsII🡪ScIII_BamHI | forward | GCTGGTGACAGCAAAAAGGATCCACCAAAGGGT |
| OL1228 | HsII🡪ScIII_BamHI | reverse | ACCCTTTGGTGGATCCTTTTTGCTGTCACCAGC |
| OL1229 | HsII🡪HsIII_∆BamHI (ScI_HsII_HsIII) | forward | GGTGACAGCAAAAA**T**GA**C**CCACCAATGGAAGCA |
| OL1230 | HsII🡪HsIII_∆BamHI (ScI_HsII_HsIII) | reverse | TGCTTCCATTGGTGG**G**TC**A**TTTTTGCTGTCACC |
| OL1234 | ∆5’BamHI HsI_ScII_HsIII | forward | AGCATAGCAATCGG**G**TCCATGGGAAAGGAA |
| OL1235 | ∆5’BamHI HsI_ScII_HsIII | reverse | TTCCTTTCCCATGGA**C**CCGATTGCTATGCT |
| OL1236 | ∆5’BamHI ScI_HsII_ScIII | forward | AGCATAGCAATCGG**G**TCCATGGGTAAAGAG |
| OL1237 | ∆5’BamHI ScI_HsII_ScIII | reverse | CTCTTTACCCATGGA**C**CCGATTGCTATGCT |
