# Supplementary material for: Eukaryotic Translation Elongation Factor 1A (eEF1A) Domain I from S. cerevisiae Is Required but Not Sufficient for Inter-Species Complementation
Source: PLoS One. 2012 Jul 30;7(7):e42338. doi: 10.1371/journal.pone.0042338 (PMC3408446; doi:10.1371/journal.pone.0042338)
Supplement: Table S3 — Plasmids used for experiments with S. Cerevisiae. (DOC) [file pone.0042338.s004.doc]

**Table S3**

| **Plasmid #** | **Construct** |
| --- | --- |
| **# 2026** | pRS314_w/o ORF |
| **# 1838** | pRS314_wtScTEF1 |
| **# 2166** | pRS314_His_6x_-ScTEF1 |
| **# 2167** | pRS314_ScTEF1-His_6x_ |
| **# 1873** | pRS314_TbHA-TEF1 |
| **# 2058** | pRS314_TbTEF1 |
| **# 2057** | pMC4_LmTEF1 |
| **# 2059** | pRS314_LmTEF1 |
| **# 2056** | pMC4_HsTEF1 |
| **# 2060** | pRS314_HsTEF1 |
| **# 2172** | pRS314_CaTEF1 |
| **# 2095** | pRS314_ScI_ScII_HsIII |
| **# 2151** | pRS314_ScI_ScII_HsIII N329K |
| **# 2096** | pRS314_ScI_ScII_TbIII |
| **# 2158** | pRS314_ScI_ScII_TbIII N329K |
| **# 2162** | pRS314_ScI_ScII_AtIII |
| **# 2161** | pRS314_ScI_ScII_AtIII frame shift ScII🡪AtIII |
| **# 2097** | pRS314_HsI_HsII_ScIII |
| **# 2156** | pRS314_HsI_HsII_ScIII N331K |
| **# 2098** | pRS314_TbI_TbII_ScIII |
| **# 2157** | pRS314_TbI_TbII_ScIII N319K |
| **# 2150** | pRS314_ScI_HsII_ScIII |
| **# 2173** | pRS314_ScI_HsII_ScIII I254T, G255S |
| **# 2164** | pRS314_HsI_ScII_HsIII |
| **# 2163** | pRS314_HsI_ScII_HsIII I256T, G257S |
| **# 2176** | pRS314_HsI_ScII_ScIII |
| **# 2177** | pRS314_HsI_ScII_ScIII N331K |
| **# 2174** | pRS314_ScI_HsII_HsIII |
| **# 2175** | pRS314_ScI_HsII_HsIII N329K |
