# Supplementary material for: Eukaryotic Translation Elongation Factor 1A (eEF1A) Domain I from S. cerevisiae Is Required but Not Sufficient for Inter-Species Complementation
Source: PLoS One. 2012 Jul 30;7(7):e42338. doi: 10.1371/journal.pone.0042338 (PMC3408446; doi:10.1371/journal.pone.0042338)
Supplement: Table S4 — Genotype of yeast strains used in this work. (DOC) [file pone.0042338.s005.doc]

**Table S4**

| **Strain #** | **Genotype and construct** |
| --- | --- |
| **#835 (TKY102)** | *MATα leu2 his4 ura3 trp1 tef2∆2 tef1::LEU2 lys2 met2 <TEF2_URA3>* |
| **# 1553** | *MATα leu2 his4 ura3 trp1 tef2∆2 tef1::LEU2 lys2 met2 <pRS314_ScI_ScII_HsIII N329K_TRP1>* |
| **# 1554** | *MATα leu2 his4 ura3 trp1 tef2∆2 tef1::LEU2 lys2 met2 <pRS314_ScI_ScII_HsIII_TRP1>* |
| **# 1588** | *MATα leu2 his4 ura3 trp1 tef2∆2 tef1::LEU2 lys2 met2 <pRS314_ScI_ScII_TbIII N329K_TRP1>* |
| **# 1555** | *MATα leu2 his4 ura3 trp1 tef2∆2 tef1::LEU2 lys2 met2 <pRS314_ScI_ScII_TbIII_TRP1>* |
| **# 1556** | *MATα leu2 his4 ura3 trp1 tef2∆2 tef1::LEU2 lys2 met2 <pRS314_ScI_HsII_ScIII_TRP1>* |
| **# 1562** | *MATα leu2 his4 ura3 trp1 tef2∆2 tef1::LEU2 lys2 met2 <pRS314_ScI_ScII_ScIII_TRP1>* |
| **# 1578** | *MATα leu2 his4 ura3 trp1 tef2∆2 tef1::LEU2 lys2 met2 <pRS314_ScTEF1-His6x_TRP1>* |
| **# 1579** | *MATα leu2 his4 ura3 trp1 tef2∆2 tef1::LEU2 lys2 met2 <pRS314_His_6x_-ScTEF1_TRP1>* |
